# The Predictive Value of Pretreatment ^18^-F-FDG-PET-CT in Locally Advanced Nasopharyngeal Cancer Patients Treated Definitively with Induction Chemotherapy Followed by Concurrent Chemo-Radiotherapy

**DOI:** 10.4172/2155-9619.1000166

**Published:** 2014-02-09

**Authors:** Hala A Zaghloul, Gehan A Khedr, Yousri Rostom, Tamer Refaat

**Affiliations:** * Department of Clinical Oncology, Faculty of Medicine, Alexandria University, Alexandria, Egypt; † Department of Radiation Oncology, Robert H. Lurie Comprehensive Cancer Center, Northwestern University Feinberg School of Medicine, Chicago, Illinois, USA

**Keywords:** ^18^F-FDG-PET-CT SUVmax, Concurrent chemoradiation, Nasopharyngeal cancer

## Abstract

**Aims:**

This study aimed to evaluate the role of pretreatment 18F-Fluorodeoxyglucose positron emission tomography (^18^F-FDG-PET-CT) as a predictor of disease-free survival (DFS), and overall survival (OS) in locally advanced nasopharyngeal carcinoma (LANPC) patients treated definitively with docetaxel-based induction chemotherapy followed by concurrent chemoradiation (CRT).

**Materials and methods:**

This is a retrospective study approved by the institutional review board and included LANC patients treated definitively and consecutively between January 2008 and December 2012 with induction chemotherapy; docetaxel, cisplatin, and 5-flurouracil (TPF) followed by CRT utilizing weekly cisplatin. All patients had baseline pretreatment ^18^F-FDG-PET-CT. We studied the association between the baseline primary tumor maximum standardized uptake value (SUVmax) and the treatment outcomes; OS and DFS.

**Results:**

The study included 70 eligible LANPC patients. The 4-year OS and DFS rates were 86.7% and 78.6%, respectively. The median OS and DFS intervals were not reached. On a univariate analysis, the 4-years DFS was significantly higher in patients with pretreatment SUVmax <8 compared versus ≥ 8 (95% vs 57.7%, P=0.002). Furthermore, DFS was significantly correlated with pretreatment T stage (P=0.01), N stage (P=0.02), treatment response (P<0.001) and treatment breaks (P<0.001). On a multivariate analysis, the SUVmax category was the only factor correlated with 4-year DFS (Hazard ratio=10.2, 95% C I 1.3-116.8, P=0.035) but not OS (P=0.085).

**Disclosure statement:**

There is no actual or potential conflict of interest with the production and publication of this work. No author has a direct or indirect commercial financial incentive associated with the publication of this article.

**Conclusion:**

This study shows that the pretreatment primary tumor ^18^F-FDG-PET-CT SUVmax is a potential independent prognostic predictor of clinical outcomes in patients with LANC treated definitively with TPF induction chemotherapy followed by CRT. Further controlled clinical trials are worthwhile.

## Introduction

Concurrent Chemoradiation (CRT) has been established as the standard treatment of locally advanced nasopharyngeal carcinoma (LANPC) based on the results of randomized clinical trials and a recent meta-analysis, which demonstrate a clear benefit of chemotherapy and radiotherapy in comparison to radiotherapy alone [1-7].

The Intergroup-0099 study demonstrated statistically significant overall survival (OS), disease free survival (DFS), and local-regional control (LRC) rate favoring CRT followed by adjuvant chemotherapy versus radiation therapy (RT) only. The study showed poor patient’s compliance in the CRT group with only 55% undergoing adjuvant treatment and notably high local-regional failure and distant metastases rates [2]. Therefore, induction chemotherapy has been an attractive treatment approach.

Furthermore, identifying reliable prognostic markers would be of ultimate importance to individualize the management of patients with LANPC. However, the pre-treatment ^18^F-FDG positron emission tomography with computed tomography (PET-CT) has been investigated as a potential tool to predict treatment outcomes in patients with head and neck cancers, the diverse tumor sites, and inconsistent results limit those studies [8-15].

This is a retrospective study that aimed to assess the role of ^18^F-FDG-PET-CT maximum standardized uptake value (SUVmax) as a reliable predictive marker, and to report the treatment outcomes, and treatment induced adverse events in LANPC patients receiving induction chemotherapy in the form of Docetaxel, Cisplatin, and 5-Fu(TPF) followed by definitive CRT.

## Patients and Methods

After obtaining the institutional review board, we reviewed charts of LANPC patients treated between January 2008 and December 2012. Eligible patients were diagnosed with LANPC stages; T1, N1-3, or T2-T4, any N according to American Joint Committee on Cancer Stage Classification System 6th Edition. All patients had baseline pretreatment PET-CT and received induction TPF chemotherapy followed by cisplatin based CRT. All patients signed informed consent. Other baseline imaging studies included computed tomography (CT), and/or Magnetic Resonance Imaging (MRI).

### Chemotherapy

Patients received with 3 cycles of induction TPF chemotherapy; docetaxel 75 mg/m^2^ and cisplatin 75 mg/m^2^ on day 1, and continuous infusion of 5-fluorouracil 750 mg/m^2^/day days 1 to 5 every 21 days. During radiation treatment, cisplatin was administered concurrently either as 40 mg/m^2^ weekly or 100 mg/m^2^ every 3 weeks. Patients were evaluated by complete physical and laboratory investigations including complete blood count and serum chemistries before each cycle of induction chemotherapy. Complete tumor assessment including physical exam, and imaging studies (CT and/or MRI) was performed after induction chemotherapy and prior to CRT.

### Radiation therapy

External beam radiation therapy (EBRT) was delivered by 3-dimensional conformal radiotherapy (3D-RT) or intensity modulated radiation therapy (IMRT) utilizing simultaneous integrated boost technique (SIB).

In patients treated with 3D-RT, each patient had three clinical-target-volumes (CTV). CTV1 included the pre-induction chemotherapy primary tumor volume and involved lymph nodes and was assigned to receive 70 Gy. CTV2 included nasopharynx, oropharynx, posterior two thirds of the anterior maxillary sinuses and non-involved upper neck nodes and received 60 Gy. CTV3 included lower non-involved neck nodes and received 54 Gy. Each CTV was expanded 5-10 mm to create the corresponding planning target volume (PTV). All patients received 1.8-2 Gy/fraction, 5 fractions/week. In patients treated with IMRT SIB, three PTVs were created (PTV1, 2, and 3) corresponding to CTVs 1, 2, and 3 in 2D-RT plans. Patient care before, during and after radiotherapy included maintaining good oral hygiene, dental care, adequate nutritional support and analgesia. Patients were assessed weekly during radiotherapy and toxicity was recorded and graded according to version 3.0 of the National Cancer Institute-Common Toxicity Criteria (NCI-CTC) [16].

### Pretreatment ^18^F-FDG-PET-CT scan

The PET scans were acquired with a PET/CT system (ECAT Exact HR+ SOMATOMA Project 10 CT Scanner /CTI PET systems (CPS), Siemens Medical Systems/Knoxville, TN). All patients fasted for at least 6 hours before PET scans and had serum glucose levels <150 mg/dL. After intravenous injection of 370 MBq ^18^F-FDG, patients were kept in the resting state in a quiet, dimly lit room for 60 min. The CT component of the consisted of a 16-slice helical scanner with a gantry port of 70 cm. Images were acquired at [11-13] bed positions. The CT acquisition was performed before the emission acquisition. CT data were used for image fusion and for generation of the CT transmission map. The patients were positioned supine with their arms placed above the head for CT acquisitions. Per our protocol, low dose CT images were obtained with oral contrast only for attenuation correction. The PET component of ECAT HR+ isbismuth germi-nate-based.Emission data were acquired for [11-13] bed positions, at 2-3 min per bed position. The field of view was from the top of the head (vertex) to the proximal thighs. Total scanning time per patient was 22-39 min. The PET/CT images were retrospectively evaluated by a radiologist and nuclear medicine physician.

### Follow up

Patients were assessed weekly during RT. Post treatment imaging studies included CT and/or MRI, and were scheduled 6-8 weeks after completion of the therapy. Tumor response was assessed according to response evaluation criteria in solid tumors (RECIST). Late radiation toxicity was assessed according to the RTOG/European Organisation for Research and Treatment of Cancer Late Radiation Morbidity Scoring Schema [17]. All patients were assessed at 3, 6, and 12 months during the first year, then every 6 months for 5 years, and then annually.

### Statistical analysis

Disease free survival (DFS) and overall survival (OS) were calculated using the Kaplan Meier analysis. Log-rank test and Cox regression analysis were performed to correlate the various clinical and pathological parameters to treatment outcomes. All analyses were performed using SPSS 13.0 package program.

## Results

This study included 70 LANPC patients who met the eligibility criteria and were treated consecutively between January 2008 and December 2012. The median age was 46 years (range 18-68) and the median follow up was 39.7 ± 10.9 months (range 14-58) for all patients. Table 1 summarizes the baseline patients and disease characteristics.

### Pretreatment ^18^F-FDG-PET-CT scan

All patients had pretreatment ^18^F-FDG-PET-CT. The PETCT SUVmax was calculated according to the following formula: SUV=tissue radioactivity concentration (MBq/g)/[injected dose (MBq)/patient weight (kg)/decay factor of ^18^F]. To minimize partial volume effects, the maximum-pixel SUV within a region of interest encompassing the tumor was used for further calculations. The median pretreatment primary tumor and lymph nodes SUVmax were 10.3 (range 3.2 to 24.3), and 8.5 (range 2.8 to 18.3) respectively.

### Chemotherapy

Sixty-three patients (90%) received 3 cycles of induction chemotherapy, and seven patients received 2 cycles of induction chemotherapy due to Grade 3 nausea and vomiting in 4 patients and febrile neutropenia in three patients.

Twenty patients (28.5%) received standard tri-weekly concomitant cisplatin (100 mg/m^2^) treatment. Seven patients of them (35%) completed 3 cycles of tri-weekly cisplatin with 25% dose reduction in 3 patients due to grade 3 mucositis, dermatitis and neutropenia, while 10 patients (50%) tolerated 2 cycles and only three patients (15%) had 1 cycle. The other 50 patients (71.4%) received concomitant cisplatin (40 mg/m^2^) on weekly basis. Of these patients, three patients (6%) received 3 weeks of weekly cisplatin, six patients (12%) had 4 weeks, 18 patients (36%) had 5 weeks, and 23 patients (46%) had 6 weeks. Of the patients who received 6 weeks of concurrent weekly cisplatin, 20% dose reduction was applied on 5 patients due to development of grade 3 adverse events. After induction chemotherapy, nine patients (12.8%) achieved complete response, 54 patients (77.1%) had partial response, and 7 patients (10%) had stable disease.

### Radiation therapy

Nineteen patients ( 27%) received IMRT utilizing SIB technique, while 51 patients (72.8%) were treated using 3D-RT. Total cumulative RT dose delivered ranged from 66 to 70 Gy (median 70 Gy).

### Treatment outcomes

All patients were assessed 6-8 weeks after definitive concurrent chemoradiation with radiologic imaging (CT and/or MRI). Sixty patients (85.7%) achieved CR and 10 patients (14.3%) had PR after treatment completion. On multiple linear regression analysis, the response achieved at the end of definitive CRT was significantly associated with tumor stage (P<0.001), nodal stage (P=0.002), treatment breaks (P<0.001) and the pretreatment PET-CT SUVmax (P=0.041). Table 2 illustrates the association between different clinicopathological factors and response.

**Figure 1:**
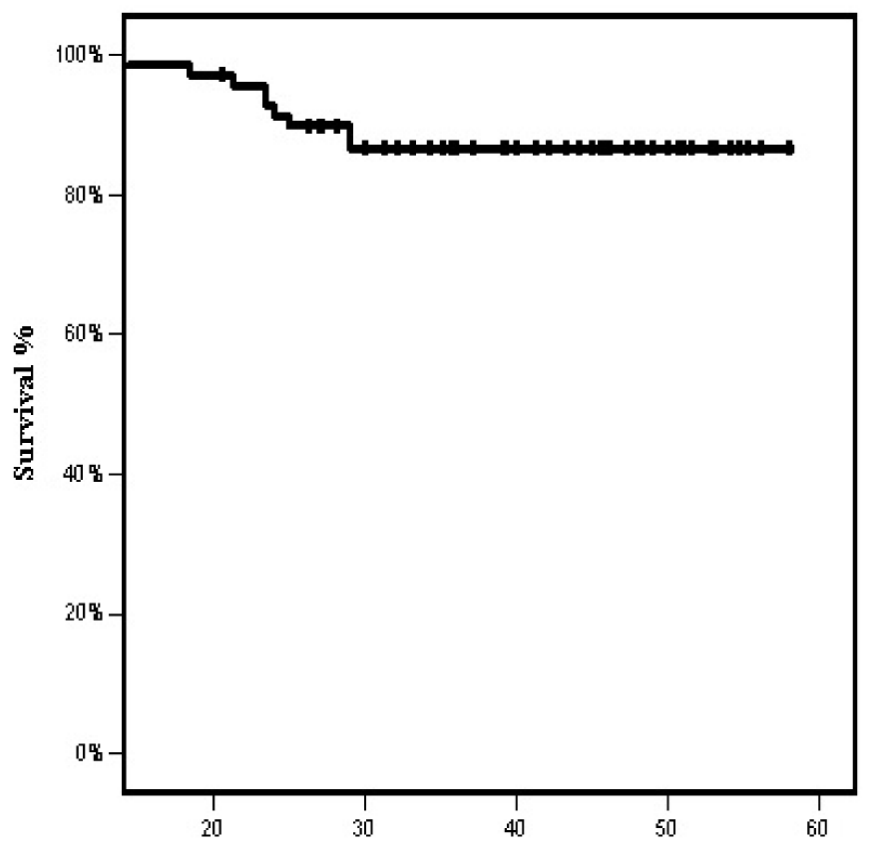
Overall survival in months.

**Figure 2:**
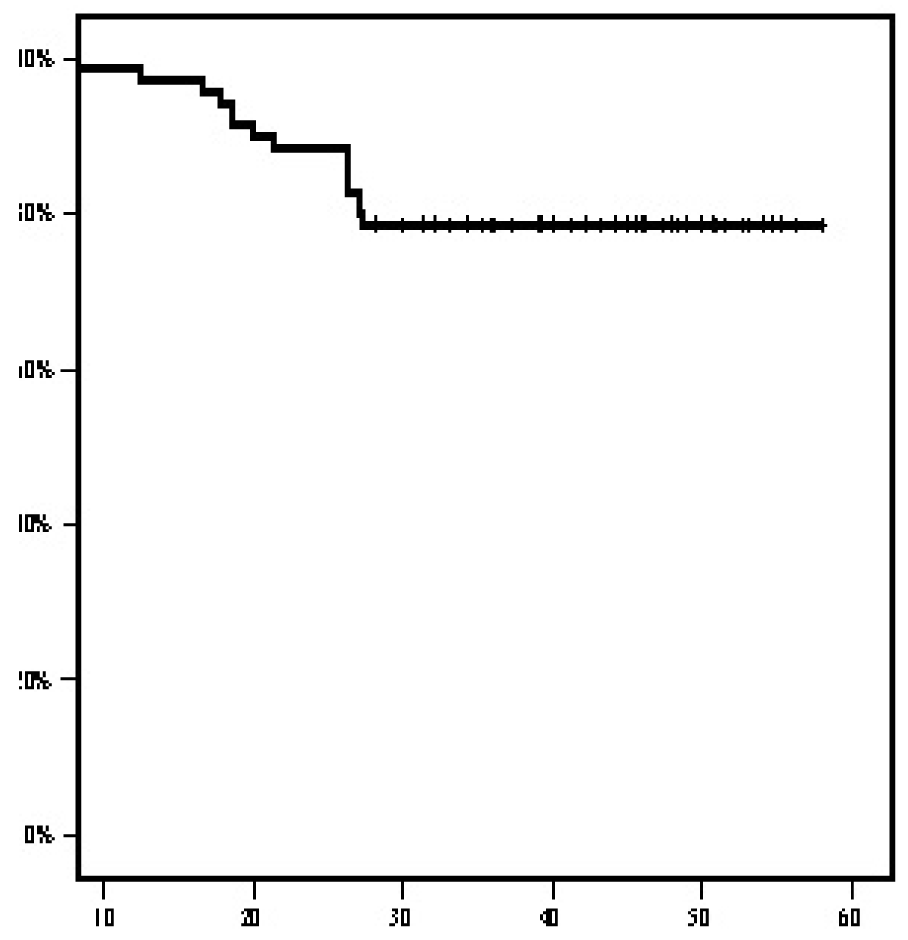
Disease free survival in months.

At a mean follow up time of 39.7 months, 13 patients (18.5%) relapsed. Three patients had local relapse only, five patients had distant metastases and five patients had both local and distant metastases. The 4-year OS and DFS rates were 86.7% and 78.6%, respectively (Figures 1 and 2). The median DFS and OS intervals were not attained.

**Figure 3:**
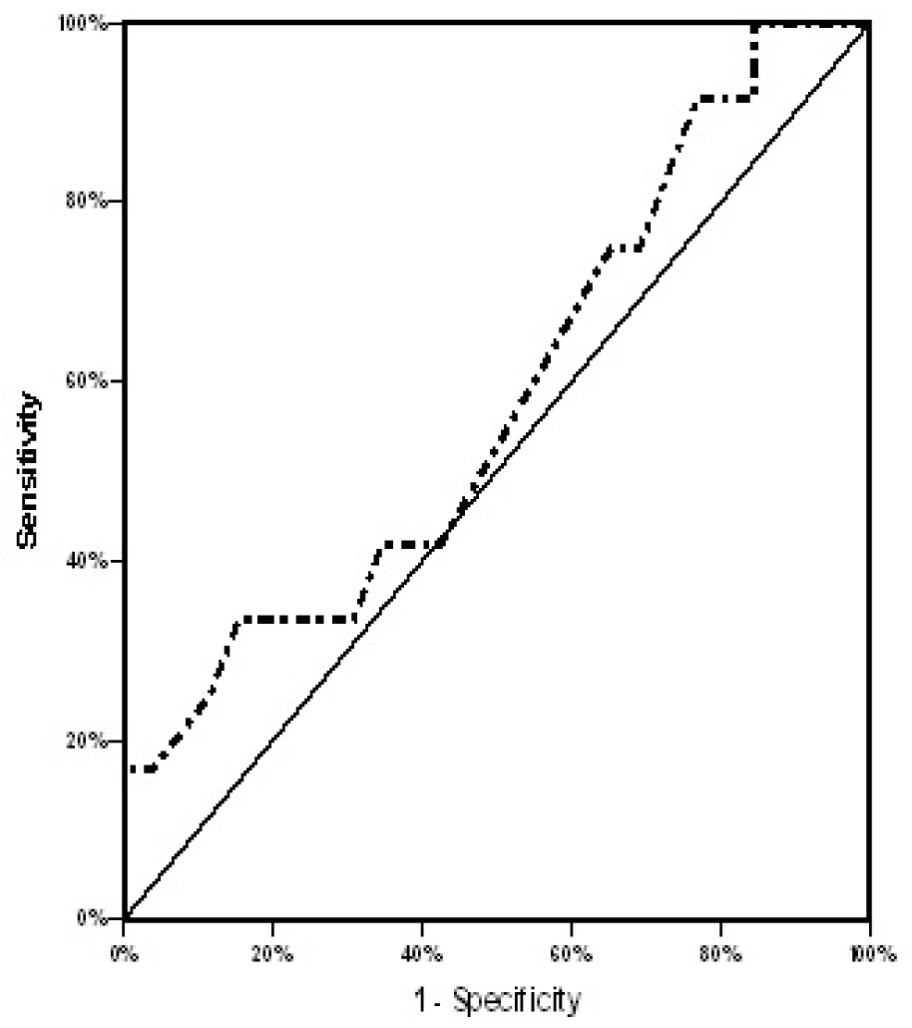
ROC curve using MaxSUV to predict DFS. Area under the curve is 0.564, and the best cut-off value is 8.0.

**Figure 4:**
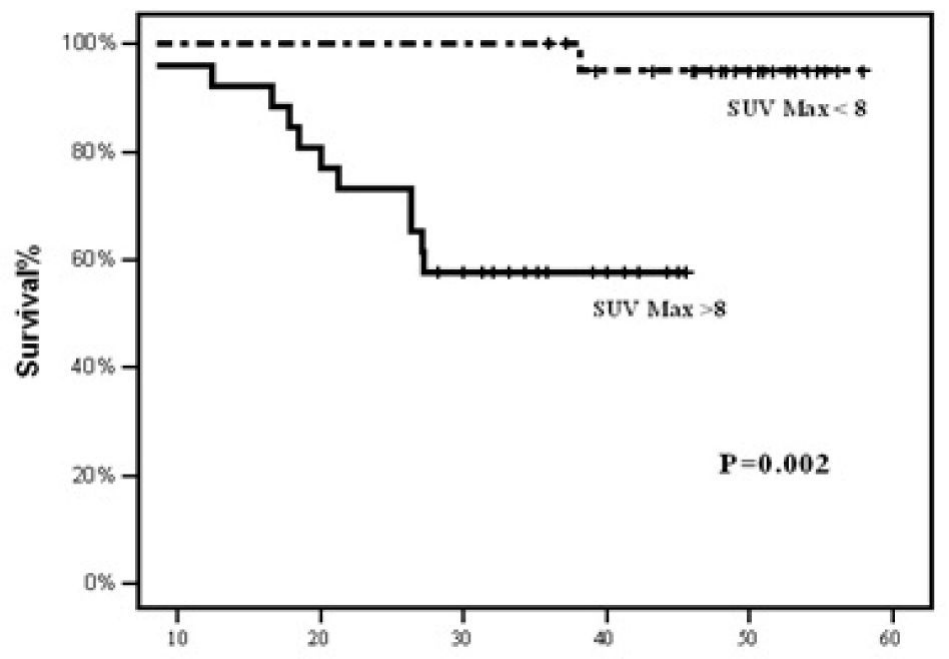
Disease free Survival (DFS) in months according to primary treatment FDG PET-CT maximal standard uptake value (SUVmax).

**Figure 5:**
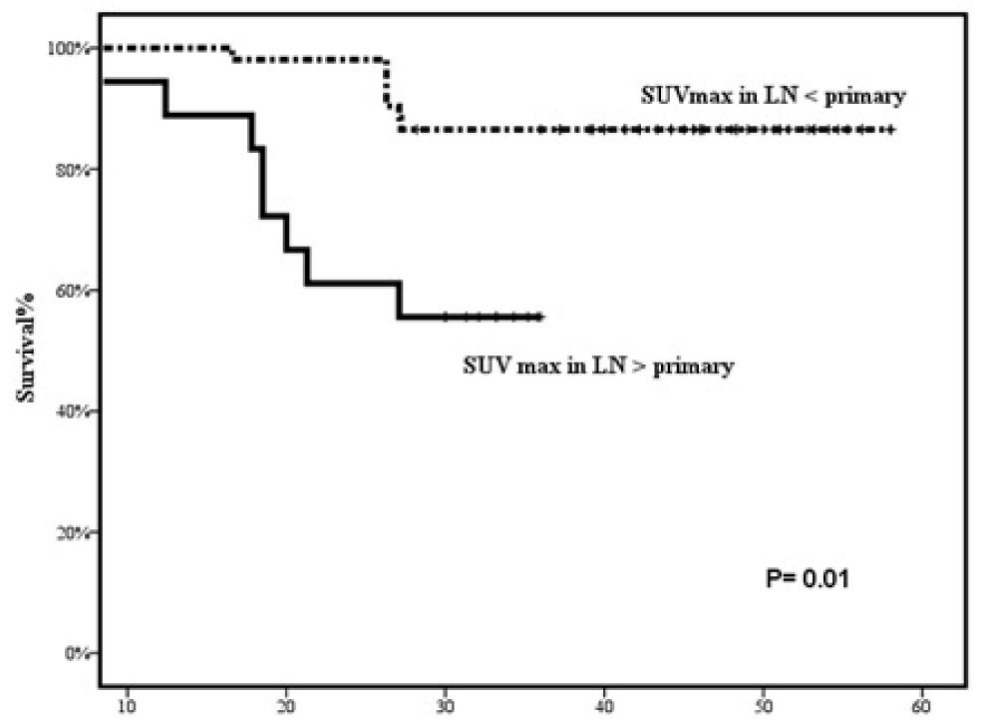
Progression free Survival in months.

Receiver Operating Characteristic (ROC) curve was used to depict the ability of SUVmax to predict prognosis. Area under the curve was 0.564, and the best cut-off value was 8.0 (Figure 3). On a univariate analysis, 4-years DFS was significantly higher in patients with pretreatment SUVmax <8 (95%) vs. ≥ 8 (57.7%) (P=0.002) (Figure 4). The 4-year DFS was also significantly associated with pretreatment T stage (P=0.01), N stage (P=0.02), treatment response (P<0.001), and treatment breaks (P<0.001). Additionally, patients with node SUVmax higher than that of the primary site had a significantly poorer DFS (55% vs 86.5%) respectively (P=0.01) (Figure 5). Moreover, patients with an SUVmax <8.0 had significantly better OS compared to those with an SUV of ≥ 8.0 (P=0.034). On a multivariate analysis, the SUVmax category was the only factor correlated with 4-year DFS (Hazard ratio=10.2, 95% C I 1.3-116.8, P=0.035). Moreover on defining the predictive value of SUVmax level in T stage categories (T1-2 and T3-4), the 4-year DFS was statistically significantly associated with SUVmax (< 8 vs. ≥ 8) (P=0.003), but not overall survival (P=0.085) when stratified by T stage (T1-T2 vs. T3-T4).

### Treatment induced adverse events

Induction chemotherapy was well tolerated, seven patients (10%) received only 2 induction cycles because of grade 3 toxicities, three (4%) of them developed grade 3 neutropenia and four (6%) patients had G3 nausea and vomiting. The most frequent acute toxicity encountered during chemoradiation was mucositis as 37 patients (53%) developed Grade 3 while only 4 patients (6%) suffered from Grade 4 mucositis, which necessitated hospitalization and discontinuation of treatment for 7-10 days.

Fourteen patients (20%) developed Grade 3 weight loss (10-19.9 Kg) while three patients (4.3%) had Grade 4 weight loss ≥ 20 kg during CRT (Table 3). Notably, the three patients who developed Grade 4 and eight of the patients who had Grade3 weight loss were initially those patients who refused the insertion of gastrostomy upfront prior the concurrent chemoradiation phase [18].

## Discussion

The role of induction chemotherapy followed by concurrent chemoradiotherapy (CRT) is a matter of outstanding interest in LANPC because of the relatively high incidence of locoregional or distant metastasis of more than 40% due to the poor patient tolerance and limited penetration of adjuvant chemotherapy after CRT [19,20]. Moreover, predicting the prognosis in those patients has become an important issue. Some reports demonstrated the value of pretreatment SUVmax as a predictive marker in patients with head and neck cancers [9-15]. However, we believe that further evidence is still required, especially among LANPC patients because the radiosensitivity, treatment strategy, and outcomes are quite different.

This study demonstrated that 3 cycles of TPF induction chemotherapy followed by CRT is a tolerable treatment modality with acceptable toxicity profile. The objective response rates (RR) were 89.6% (complete response (CR) was 12.5% and partial response (PR) was 77.1%) after induction TPF and 100% (CR in 85.7% and PR in 14.3%) after treatment completion. These results compare favorably with previous reports on induction chemotherapy. Bae et al. reported objective RR of 97% after induction chemotherapy (CR in 15.2% and PR in 81.8%) and 97% (CR in 69.7% and PR in 27%) after CRT (18). Similarly, Ekenel et al. reported objective RR of 87% and CR of 12% after induction and 100% objective RR with 95% CR after CRT [19].

In our study, the 4-year OS and PFS rates were 86.7% and 78.6%, respectively .The median DFS and OS intervals were not attained. Comparable survival rates have been reported. Bae et al. treated 32 LANC patients with TPF followed by CRT, PFS and OS rates were 75% and 86% respectively [18]. Hellenic Cooperative Oncology Group Study, evaluated induction cisplatin, epirubicin and paclitaxel chemotherapy to 47 patients, the 1-year OS was 93.5% and the 2-year PFS was 62% [20]. Ekenel et al. reported 94.9% and 84.7% 3 year OS and PFS, respectively [19]. Hiu et al. showed a clear OS benefit with this induction strategy, the 3-year PFS and OS were 88.2% and 94.1%, respectively [21].

Kong et al. reported the results of phase II trial on induction TPF followed by CRT using weekly cisplatin, the overall RR after RT was 90.2% and the 1-year OS was 100% [22].

The pretreatment ^18^F-^18^F ^18^F FDG-PET-CT-CT-CT, in addition to being used for the diagnostic work-up of patients with LANPC, ^18^F-FDG-PET-CT uptake, as measured by maximal SUV, showed a statistically significant association with DFS rate in NPC patients treated with CCRT [11]. In agreement with previous findings, our study demonstrated that LANPC patients treated by induction TPF chemotherapy followed by CRT with a base line SUVmax <8.0 had a significantly superior DFS (95% vs 57%) (P=0.002) respectively compared to those with SUVmax ≥ 8. Similarly, lee et al. confirmed that NPC with an SUVmax <8.0 had higher DFS than patients with SUVmax ≥ 8 (91% vs 51%) respectively, P=0.007 [11]. On a multivariate analysis, the SUVmax category was the only factor correlated with 4-year DFS (Hazard ratio=10.2, 95% C I 1.3-116.8, P=0. 035). Similarly, Liu et al. concluded on multivariate analysis that the SUVmax was the only significant variable for 5-year LFFS (p=0.017) and DFS (p=0.000) [14]. However, other studies demonstrated that the pretreatment FDG uptake is the only significant variable predicting survival and recurrence on multivariate analysis in LANPC patients, most of them were not specifically scrutinizing on patients receiving induction followed by CRT [8-15]. Our study also demonstrated that the 4-year DFS, and OS in T-stage (T1-2 versus T3-4) stratified by SUVmax level (<8 vs. ≥ 8) were significantly associated with better DFS (P=0.003), but not for overall survival (P=0.085) favoring the SUVmax <8. In contrast, Xie et al. demonstrated that the pretreatment SUVmax significantly affects the OS and DFS [15]. A possible explanation for our results not showing impact on OS when stratified by stage is that most of the patients with local-regional recurrence have survived after salvage treatment.

The most commonly encountered acute toxicities during induction chemotherapy were Grade 3 neutropenia, nausea and vomiting in 4% and 6% of patients respectively, which were uncomplicated and manageable. The most frequent acute toxicity encountered during CRT was mucositis as 37patients (53%). Moreover, fourteen patients (20%) developed Grade 3 weight loss (10-19.9 Kg) while three patients (4.3%) had Grade 4 weight loss ≥ 20 kg during the CRT phase. Similarly, Bae et al. reported that febrile neutropenia (9.1%), and nausea (9.1%) as the most notable grade 3 and 4 toxicities during induction chemotherapy phase, while mucositis (39.4%), fatigue (15.2%), and nausea (9.1%) were the most common grade 3 and 4 toxicities during CRT [18]. Additionally, Ekenel et al. reported that TC induction chemotherapy followed by CRT was well tolerated with a 10% rate of Grade 3/4 hematologic toxicity. There was no treatment related deaths [19]. Consequently, we believe that induction chemotherapy has an evolving role in the management of locally advanced NPC and is associated with tolerable toxicity profile. Pretreatment PET-CT SUVmax is a potential prognostic marker for LANPC patients receiving induction chemotherapy followed by CRT.

## Conclusion

The pretreatment primary tumor PET-CT SUVmax is a potential independent prognostic predictor of clinical outcomes in patients with LANPC treated with TPF induction chemotherapy followed by CRT. A high ^18^F-FDG uptake (SUVmax ≥ 8) may indicate poor outcome in such patients.

## Figures and Tables

**Table 1:** Patients and disease characteristics at baseline (N=70).

Characteristic	No. of Patients	%
**Age, years**		
Median	46	
Range	18-68	
**Sex**		
Male	52	75%
Female	18	25%
**Pathological Subtype**		
Non keratinizing	13	18.5%
Undifferentiated	50	71.4%
Basaloid	7	10.1%
**T stage**		
T1	7	10.1%
T2	26	37.1%
T3	29	41.4%
T4a,b	8	11.4%
**N stage**		
N0	18	25.7%
N1	39	55.7%
N2	10	14.3%
N3	3	4.3%
**Stage group**		
II	18	25.7%
III	43	61.4%
IVA,B	9	12.9%

**Table 2:** Association between response and other clinicopathological factors.

Factor	Patients no		P Value
	CR	PR	
**Sex**			**0.889**
Male	45 (64.2%)	7 (10.2%)	
Female	15 (21.4%)	3 (4.2%)	
**Pathological subtype**			**0.133**
Non keratinizing	9 (12.9%)	4 (5.7%)	
Undifferentiated	45 (64.3%)	5 (7.1%)	
Basaloid	5 (7.1%)	2 (2.9 %)	
**Baseline T stage**			***<0 .001***
T1	7 (10.1%)		
T2	26 (37.1%)		
T3	25 (35.7%)	4 (5.7%)	
T4	3 (4.3%)	5 (7.1%)	
**Baseline N stage**			***0.002***
N0	18 (25.7%)		
N1	34 (48.6%)	5 (7.1%)	
N2	9 (12.9%)	1 (1.4%)	
N3	3 (4.3%)		
**Radiation Technique**			***0.412***
3DCRT	48 (68.6%)	3 (4.2%)	
IMRT	15 (21.4%)	4 (5.8%)	
**Concurrent Chemo-radiation** **Break**			***<0 .001***
<7 Days	56 (80%)	4 (5.7%)	
>7 Days	3 (4.3%)	7 (10 %)	
**Pretreatment Pet- CTSUVmax** **primary**			***041***
<8	32 (45.7%)	6 (8.6%)	
≥ 8	28 (40%)	4 (5.7%)	

**Table 3:** Treatment related Grade 3&4 acute toxicities.

Toxicity	Induction chemotherapy No. of patients (%)		Chemoradiation No. of patients (%)	
	Grade 3	Grade 4	Grade 3	Grade 4
Mucositis	0	0	37 (53%)	4 (5.7%)
Weight loss	0	0	14 (20%)	3 (4.3%)
Esophagitis	0	0	10 (14%)	0
Nausea/vomiting	4 (5.7%)	0	6 (8.5%)	0
Anemia	0	0	1 (1.4%)	0
Neutropenia	2 (2.8%)	0	4 (6%)	0
Thrombocytopenia	0	0	3 (4.3%)	0
Dermatitis	0	0	28 (40%)	0

## References

[1] Chan AT, Leung SF, Ngan RK, Peter TeoML, Lau WH (2005). Overall survival after concurrent cisplatin radiotherapy compared with radiotherapy alone in locoregionally advanced nasopharyngeal carcinoma. J Natl Cancer Inst.

[2] Al-Sarraf M, LeBlanc M, Giri PG, Fu KK, Cooper J (2008). Chemoradiotherapy versus radiotherapy in patients with advanced nasopharyngeal cancer: phase III randomized Intergroup study 0099. J Clin Oncol.

[3] Chen Y, Liu MZ, Liang SB, Zong JF, Mao YP (2008). Preliminary results of a prospective randomized trial comparing concurrent chemoradiotherapy plus adjuvant chemotherapy with radiotherapy alone in patients with locoregionally advanced nasopharyngeal carcinoma in endemic regions of china. Int J Radiat Oncol Biol Phys.

[4] Chua DT, Ma J, Sham JS, Hai-Qiang Mai, Damon Choy TK (2005). Long-term survival after cisplatin-based induction chemotherapy and radiotherapy for nasopharyngeal carcinoma: a pooled data analysis of two phase III trials. J Clin Oncol.

[5] Langendijk JA, Leemans CR, Buter J, Berkhof J, Slotman BJ (2004). The additional value of chemotherapy to radiotherapy in locally advanced nasopharyngeal carcinoma: a meta analysis of the published literature. J Clin Oncol.

[6] Baujat B, Audry H, Bourhis J, Chan AT, Onat H (2006). Chemotherapy in locally advanced nasopharyngeal carcinoma: an individual patient data metaanalysis of eight randomized trials and 1753 patients. Int J Radiat Oncol Biol Phys.

[7] Lee AW, Tung SY, Chua DT, Ngan RK, Chappell R (2010). Randomized trial of radiotherapy plus concurrent-adjuvant chemotherapy vs radiotherapy alone for regionally advanced nasopharyngeal carcinoma. J Natl Cancer Inst.

[8] Eisenhauera E, Therasseb P, Bogaertsc J, Schwartzd LH, Sargente D (2009). New response evaluation criteria in solid tumours: Revised RECIST guideline (version 1.1). Eur J Cancer.

[9] Allas AS, Slosman DO, Ani TK, Allaoua M, Lehmann W (2004). Prediction of outcome in head-and-neck cancer patients using the standardized uptake value of 2-[18F]fluoro-2-deoxy-d glucose. Int J Radiation Oncology Biol Phys.

[10] Yen TC, Lin CY, Wang HM, Huang SF, Liao CT (2006). ^18^FFDG-PET for evaluation of the response to concurrent chemoradiation therapy with intensity-modulated radiation technique for stage T4 nasopharyngeal carcinoma. Int J Radiation Oncology Biol Phys.

[11] Lee SW, Yuhl NS, Im KC, Kimc Jae Seung, Choi Eun Kyung (2008). Prediction of prognosis using standardized uptake value of 2-[18F]fluoro-2-deoxy-D-glucose positron emission tomography for nasopharyngeal carcinomas. Radiother Oncol.

[12] Ma BB, Mo FK, Chan AT, Hui EP, Leung SF (2008). The prognostic significance of tumor vascular invasion and its association with plasma Epstein-Barr virus DNA, tumor volume and metabolic activity in locoregionally advanced nasopharyngeal carcinoma. Oral Oncol.

[13] Chan SC, Chang JT, Wang HM, Lin CY, Ng SH (2009). Prediction for distant failure in patients with stage M0 nasopharyngeal carcinoma:The role of standardized uptake value. Oral Oncol.

[14] Liu WS, Wu MF, Tseng HC, Liu JT, Weng JH (2012). The role of pretreatment FDG-PET in nasopharyngeal carcinoma treated with intensity-modulated radiotherapy. Int J Radiat Oncol Biol Phys.

[15] Xie P, Yue JB, Fu Z, Feng R, Yu JM (2010). Prognostic value of ^18^F-FDG PET/ CT before and after radiotherapy for locally advanced nasopharyngeal carcinoma. Ann Oncol.

[16] Cancer Therapy Evaluation Program Common terminology criteria for adverse events.

[17] Cox JD, Stetz J, Pajak TF (1995). Toxicity criteria of the Radiation Therapy Oncology Group (RTOG) and the European Organization for Research and Treatment of Cancer (EORTC). Int J Radiat Oncol Biol Phys.

[18] Bae WK, Hwang JE, Shim HJ, Cho SH, Lee JK (2010). Phase II study of docetaxel, cisplatin, and 5-FU induction chemotherapy followed by chemoradiotherapy in locoregionally advanced nasopharyngeal cancer. Cancer Chemother Pharmacol.

[19] Ekenel M, Keskin S, Basaran M, Bavbek E, Ozdemir C (2010). Clinical outcomes in patients with locally advanced nasopharyngeal cancer treated with neoadjuvant docetaxel and cisplatin followed by radiation treatment and concomitant cisplatin. J Clin Oncol Suppl.

[20] Fountzilas G, Tolis C, Kalogera-Fountzila A, Karanikiotis C, Bai M (2005). Induction chemotherapy with cisplatin, epirubicin, and paclitaxel (CEP), followed by concomitant radiotherapy and weekly paclitaxel for the management of locally advanced nasopharyngeal carcinoma. A Hellenic Cooperative Oncology Group phase II study. Strahlenther Onkol.

[21] Hui EP, Ma BB, Leung SF, King AD, Mo F (2009). Randomized phase II trial of concurrent cisplatin-radiotherapy with or without neoadjuvantdocetaxel and cisplatin in advanced nasopharyngeal carcinoma. J Clin Oncol.

[22] Kong L, Zhang YW, Hu CS, Guo Y (2010). Neoadjuvant chemotherapy followed by concurrent chemoradiation for locally advanced nasopharyngeal carcinoma. Chin J Cancer.

